# Epidemiology of interpersonal violence among Mexican children and adolescents: a national analysis of injury data from public hospitals from 2015 to 2022

**DOI:** 10.1186/s12889-025-22990-z

**Published:** 2025-05-09

**Authors:** Magdalena Gruendl, Diana D. del Valle, Letícia Nunes Campos, Theoneste Nkurunziza, Taylor Wurdeman, Tanujit Dey, Arturo Cervantes Trejo, Stefanie J. Klug, Tarsicio Uribe-Leitz

**Affiliations:** 1 Chair of Epidemiology, TUM School of Medicine and Health, Technical University of Munich, Am Olympiacampus 11, 80809 Munich, Germany; 2 Program in Global Surgery and Social Change, Harvard Medical School, Boston, USA; 3 Faculty of Medical Sciences, University of Pernambuco, Recife, Brazil; 4 The Center for Surgery and Public Health, Brigham and Women’s Hospital, Boston, USA; 5 Faculty of Health Sciences, Anahuac University, Anahuac, Mexico; 6 Department of Plastic and Oral Surgery, Boston Children’s Hospital, Boston, USA

**Keywords:** Interpersonal violence, IPV, Children, Adolescents, Mexico

## Abstract

**Introduction:**

Interpersonal violence (IPV) among children and adolescents represents a significant global public health problem. While Mexico has recorded an increase in IPV, its distribution and management remain understudied. We aim to investigate the epidemiology of IPV cases among children and adolescents in Mexico.

**Methods:**

This retrospective registry-based analysis used a nationwide injury dataset (*Lesiones)* from the Mexican Ministry of Health. We included medical records of IPV victims aged 0 to 17 years who presented at public health facilities in Mexico from 2015 to 2022. We used stratified descriptive statistics to summarize the distribution, management, and outcomes of IPV. Categorical variables were compared between male and female victims, as well as across age categories, using chi-square tests. Additionally, we generated a heatmap to visually represent the average IPV cases per 100,000 children and adolescents across Mexican states.

**Results:**

Among 116,287 IPV victims, 36,385 (31.3%) were male and 79,902 (68.7%) female. The majority were aged 15–17 years (*n* = 62,616; 53.8%), followed by those aged 10–14 years (*n* = 34,234; 29.4%), 5–9 years (*n* = 12,219; 10.5%), and under 5 years (*n* = 7,218; 6.2%). Most had a secondary education (*n* = 32,509; 28.0%), and literacy levels were high, with 86,858 (74.7%) reported as literate. Among female victims, 11,207 (14.0%) were pregnant at the time of the IPV incident. Among all victims, physical abuse (*n* = 39,155; 33.7%) was the most common form of violence, followed by mental abuse (*n* = 38,759; 33.3%) and sexual abuse (*n* = 38,373; 33.0%). Among males, 73.5% (*n* = 26,743) were affected by physical abuse, whereas among females, 44.4% (*n* = 38,373) experienced sexual abuse. Family violence accounted for 57.1% (*n* = 66,407) of all medical records. The states with the highest average IPV cases per 100,000 children and adolescents were Guanajuato (83.8) and Chihuahua (80.0). Most aggressors were male (*n* = 76,909; 66.1%).

**Conclusion:**

This study analyzed IPV cases among children and adolescents in Mexico. Findings highlight the need for multi-faceted, age- and gender-specific interventions. Strengthening laws and policies, with a focus on reporting, enforcement, and mandatory training, is essential to protect children and adolescents from IPV.

**Supplementary Information:**

The online version contains supplementary material available at 10.1186/s12889-025-22990-z.

## Summary of findings and public health implications

This study provides a comprehensive analysis of IPV cases among children and adolescents in Mexico, revealing critical patterns at multiple levels. At the individual level, adolescent girls are disproportionately affected by mental and sexual abuse. Relationship dynamics indicate that IPV predominantly occurs in family settings, reinforcing the role of power imbalances within households. Community-level disparities suggest that socio-economic factors contribute to IPV prevalence, while at the societal level, entrenched gender norms and weak law enforcement perpetuate violence.

Key findings highlight gender disparities, with females constituting 68.7% of IPV-related medical records and most aggressors being male (66.1%). While physical abuse is more common among males, mental and sexual abuse disproportionately affect females. Notably, sexual abuse is highly prevalent among girls aged 5–14, underscoring the urgent need for protective measures and educational interventions. The study also identifies residential areas as the primary locations for IPV incidents, with lockdowns exacerbating exposure to violence.

Geographic variations in IPV case rates, with notably high numbers in states like Guanajuato and Chihuahua, suggest the impact of socio-economic inequalities.. Although IPV cases declined during the pandemic, this likely reflects underreporting rather than a true reduction in violence.

These findings have significant public health implications, emphasizing the need for multi-level interventions. Policy measures should include gender-sensitive prevention strategies, improved access to mental health services, culturally tailored community programs, and strengthened legal enforcement. Future research should focus on evaluating intervention effectiveness and developing more comprehensive legal frameworks to protect vulnerable populations.

## Background

Interpersonal violence (IPV) against children and adolescents is a significant public health issue with severe physical, psychological, and socio-economic consequences. The World Health Organization (WHO) defines IPV as harm inflicted by one individual upon another. IPV encompasses physical (e.g., hitting, kicking, pushing), sexual (e.g., forced sexual acts, sexual coercion), and mental abuse (e.g., threats, controlling behaviors) [1]. IPV can occur in various contexts, including familial relationships, peer interactions, and encounters with strangers [2]. Acts of interpersonal violence can further be classified into family or partner violence, which includes child maltreatment and intimate partner violence, with homicide being the most extreme form, and community violence, encompassing acts such as bullying and assault by acquaintances or strangers, and violence in institutional settings like schools and workplaces [3, 4]. Addressing this issue aligns with Sustainable Development Goal (SDG) 5: Achieve gender equality and empower all women and girls, which includes the aim to eliminate all forms of violence against women and girls in the public and private spheres. [5].

Globally, as of 2019, the all-age incidence rate of IPV was 413.4 per 100,000 population resulting in a mortality rate of 5.2 per 100,000 population [6]. The incidence rates were 203.1 per 100,000 population for children under 5 years and 185.9 per 100,000 for those aged 5–9 years. These rates increased to 317.6 per 100,000 in the 10–14 age group and 610.1 per 100,000 among individuals aged 15–19 years. [6]. Of all-age global IPV deaths, 24.8% occurred in adolescents (10–24 years), with a significantly higher rate in males than in females [7]. However, females were disproportionately affected by sexual violence, with more than twice as many disability-adjusted life years (DALYs) as males (42.8 vs. 17.5 per 100,000) [7]. A comprehensive report by the United Nations Office on Drugs and Crime (UNODC) analyzed homicide data from 2008 to 2017, revealing that approximately 205,153 children aged 0 to 14 years were killed globally due to homicide [8]. Worldwide, 1 billion children have experienced IPV [9]. Case estimates indicate that at least 50% of children in Asia, Africa, and Northern America experienced violence in the past year [10]. Additionally, the Pan American Health Organization (PAHO) reports that an estimated 58% of children in Latin America experienced physical, sexual and/or emotional abuse in the past year [11].

Violence has profound health and economic repercussions. In addition to a substantial increase in mortality and disability, exposure to IPV, including intimate partner violence, significantly elevates the risk of depression, post-traumatic stress disorder, suicide, somatic disorders, and adverse reproductive health outcomes [12, 13]. Moreover, the socio‐economic consequences of adolescent violence are considerable and often enduring, manifesting as educational setbacks, a heightened risk of unemployment, and a sustained likelihood of poverty [14]. Economically, the impact is equally staggering. In 2021, the cost of crime and violence in Mexico was estimated at 192 billion USD, equivalent to 14.6% of the national Gross Domestic product (GDP). Projections indicate that by 2030, these costs could rise to as much as 19.1% of the GDP [15].

Despite being preventable, IPV remains a leading cause of injury and death among young populations globally, particularly in low- and middle-income countries (LMICs) where resources for prevention and treatment are limited [16]. The UN Declaration on the Elimination of Violence Against Women (1993) defines violence against women as a human rights violation [17]. In accordance with international human rights law, governments are required to condemn violence without using cultural or religious justifications, implement policies and legislation to prevent violence, ensure access to justice and remedies for victims, provide protective and support services, and offer gender-sensitive training to judicial and law enforcement officers [18]. In May 2016, a World Health Assembly resolution endorsed the first-ever WHO Global Plan of Action on strengthening the role of health systems within a national multisectoral response to address IPV, particularly against women, girls, and children [19]. Patriarchal structures and societal acceptance play a significant role in perpetuating IPV [20]. Children and adolescents raised in patriarchal societies often internalize aggressive behaviors, with boys being socialized to equate masculinity with dominance and control. This fosters environments where violence is accepted to resolve conflicts or assert authority, increasing the prevalence of IPV [20]. Furthermore, the societal acceptance of violence as a normative behavior, rooted in patriarchal ideologies, reinforces cycles of abuse. For example, children and adolescents who witness domestic violence are more likely to engage in violent behaviors themselves, perpetuating patterns of IPV across generations [21]. In patriarchal cultures, aggressive behavior is often seen as an acceptable expression of masculinity, which normalizes male aggression towards women and positions it as a natural outcome of socialization processes [22].

Mexico, a middle-income country in North America, with over 126 million inhabitants as of 2022 [23], has 31 states and a federal government. During the ongoing epidemiological transition, the health burden attributable to non-communicable diseases and injuries in the country has significantly increased, accounting for less than one-third of all-cause mortality in 1950 but nearly 90% by 2010 [24]. Between 1990 and 2019, IPV increased by 84.1% across all age groups in Mexico [15]. The PAHO identified increased rates of years lived with disability (YLD) due to IPV between 2000 and 2019, observing a rate of 64.7 per 100,000 Mexicans, higher among women than men (86.5 and 45.7 per 100,000 respectively) in 2019 [25]. Furthermore, there has been an increasing burden among women, with a four-point rise in total lifetime violence observed between 2016 and 2021 (from 66.1% to 70.1%) [26]. Regarding children, IPV accounted for 10.2% of deaths among 5 to 14-year-olds in Mexico [27]. Among adolescents aged 15 to 19 years in 2019, IPV was the leading cause of disability-adjusted life years (DALYs) in Mexico [1].

Despite Mexico enacting laws to protect children and adolescents against violence, the enforcement of such laws remains inadequate [2]. Moreover, little is known about the specific characteristics and patterns of IPV cases in Mexico, particularly among children and adolescents. This study aims to describe the epidemiology of IPV cases among children and adolescents in Mexico between 2015—2022. To analyze the multifaceted nature of IPV among children and adolescents in Mexico, this study adopts the Social-Ecological Model (SEM), which identifies factors influencing IPV at the individual, relational, community, and societal levels [28]. This framework, which is also commonly used by the World Health Organization Violence Prevention Unit [29], allows for a comprehensive understanding of IPV beyond individual behaviors, considering broader structural determinants.

## Methods

### Study design

This retrospective nationwide registry-based analysis included medical records of all children and adolescents aged 0 to 17 years old who were victims of IPV and treated at public hospitals in Mexico between January 2015 and December 2022. For each victim, information on their aggressor was available. The data include both the pre-COVID pandemic period (before the pandemic's global impact) and the pandemic years during which COVID-19 significantly affected healthcare systems and societal functioning including lockdown periods.

### Study setting

The Mexican healthcare system is highly fragmented but can be broadly divided into two components: The public and private healthcare sectors. Approximately 1.3% of the Mexican population can access private services [30], meaning that most citizens receive healthcare at public institutions, which are primarily funded by the federal government. The public healthcare system also includes social security institutions, such as the Mexican Social Security Institute (IMSS), which provides services to workers and their families employed in the formal sector of the economy [30]. The rates of uninsured individuals vary, ranging from 25.0% to 40.1% of the population, as reported in previous studies [31, 32]. The pediatric population in our study received care in the Mexican public healthcare sector.

### Data sources

We obtained demographic and clinical data from the *Lesiones (Injury)* database for the years 2015–2022. The *Lesiones* database is a publicly available national injury database that contains health data from public hospitals managed by the Mexican Ministry of Health (Secretaría de Salud), accounting for roughly one-third of all hospitals in the country [33]. To enhance our analysis, we supplemented this dataset with age-specific population data from Mexico’s 2020 census, obtained from the National Institute of Statistic and Geography *(INEGI)* [26]. The INEGI data was used to calculate the average number IPV cases per 100,00 children and adolescents.

### Study population

All children and adolescents aged 0 to 17 years old who were victims of IPV, classified either as family or non-family violence, were included. The World Health Organization defines violence against children as all forms of violence against people under 18 years old [19]. Victims of unintentional traffic accidents, self-inflicted injuries, human trafficking or those with unspecified circumstances as well as victims, whose age or sex data were missing, were excluded.

### Study outcome and variables

The primary outcome of the study was the number of IPV cases, defined as incidents of physical, sexual, or mental abuse (Supplementary Table 1). To analyze the demographic characteristics of IPV victims, several categorical variables were examined, including sex (male and female), children’s age (less than 5 years, 5–9 years, 10–14 years, and 15–17 years, as grouped by the National Institute of Child and Human Development, USA [34]. Additionally, education level was categorized as not educated, primary education, secondary education, beyond secondary education and missing, while literacy status, indigenous background, disability status, and pregnancy status were recorded as dichotomous (yes or no) variables. This allowed for a comprehensive understanding of the socio-demographic context of IPV victims. The forms of IPV were categorized into physical, sexual, and mental violence using ICD-10 codes recorded in the *Lesiones* dataset. IPV was classified based on the type of violence as family violence (occurring within familial relationships) or non-family violence (occurring between unrelated individuals such as acquaintances or strangers). The analysis further considered the location of the incident, grouping it into residential areas (private homes), schools, recreational locations, transportation, commercial locations, workplaces, and unspecified. Key event-related variables included whether the incident occurred on a festive day, whether the violence was recurrent, and whether the victims received prehospital medical care known to improve outcomes in severe trauma cases. To examine healthcare responses, the study assessed the medical services provided that were categorized into external consultations, hospitalizations, emergency consultations, specialized violence care services and other. The roles of healthcare providers—physicians, psychologists, and social workers—were also analyzed, alongside the victims’ post-treatment destinations, which included returning home, transfer to another medical unit, specialized violence care services, external consultations, subsequent hospitalization, deceased and other. Characteristics of aggressors, such as sex (male, female, unspecified), age (less than 18, 18–30, 31–50, 51–70, more than 70, or unspecified), and their relationship with the victim (biological parent, spouse/partner/boyfriend, other relative, stepfather/mother, non-family acquaintance, other and unspecified) were included to provide a holistic understanding of the dynamics and contexts of IPV cases. The inclusion of the Mexican state variable enabled the assessment of regional variations in IPV cases.

### Statistical analysis

We conducted stratified analysis to explore the demographics and clinical characteristics of the victims of IPV and the characteristics of their aggressors. Categorical variables were summarized using frequencies and percentages. We used the Chi-square test to compare proportions between male and female groups. We utilized SankeyMATIC to create a Sankey flow diagrams for detailed visualization of the distribution of forms of violence across age and sex categories. We generated a heatmap to visually represent the average IPV cases per 100,000 children and adolescents across Mexican states. Geographic shapefiles of Mexican states were sourced from the University of Texas Libraries'geodata repository (Format: Shapefile—University of Texas Libraries GeoData Search Results). Incidence rates were calculated using data for children and adolescents aged 0–17 years, extracted from *INEGI* [26]. STATA V18 (College Station, Texas, USA) was used for statistical analysis.

### Ethical considerations

This project was submitted to the Institutional Review Board and considered non-human subjects research (protocol #IRB23-0178) since we analyzed secondary de-identified data that are publicly available.

## Results

Among the 116,287 IPV victims, 36,385 (31.3%) were male and 79,902 (68.7%) female (Table 1). The majority were aged 15–17 years (*n* = 62,616; 53.8%), followed by those aged 10–14 years (*n* = 34,234; 29.4%), 5–9 years (*n* = 12,219; 10.5%), and under 5 years (*n* = 7,218; 6.2%). Most had a secondary education (*n* = 32,509; 28.0%), and literacy levels were high, with 86,858 (74.7%) reported as literate. 2.0% (*n* = 2,274) self-identified as Indigenous, and 1.3% (*n* = 1,472) reported having disabilities. Among female victims, 11,207 (14.0%) were pregnant at the time of the IPV incident.

**Table 1:** Demographic characteristics of the IPV victims (*n* = 116,287)

	**Male** **36,385 (31.3%)**		**Female** **79,902 (68.7%)**		**Total** **116,287 (100.0%)**		***p*-value**
	n	**%**	n	**%**	n	**%**	
Age categories							
Under 5 years old	3,017	8.3	4,201	5.3	7,218	6.2	< 0.001
5–9 years old	5,200	14.3	7,019	8.8	12,219	10.5	
10–14 years old	9,136	25.1	25,098	31.4	34,234	29.4	
15–17 years old	19,032	52.3	43,584	54.5	62,616	53.8	
Level of education							
Not educated	8,796	24.2	14,970	18.7	23,766	20.4	< 0.001
Primary education	4,348	11.9	11,527	14.4	15,875	13.7	
Secondary education	6,706	18.4	25,803	32.3	32,509	28.0	
Beyond secondary education	51	0.1	206	0.3	257	0.2	
Missing	16,484	45.3	27,396	34.3	43,880	37.7	
Literacy status							
Yes	26,321	72.3	60,537	75.8	86,858	74.7	< 0.001
No	6,221	17.1	11,507	14.4	17,728	15.2	
Missing	3,843	10.6	7,858	9.8	11,701	10.1	
Indigenous status							
Yes	499	1.4	1,775	2.2	2,274	2.0	< 0.001
No	15,302	42.1	45,306	56.7	60,608	52.1	
Missing	20,584	56.6	32,821	41.1	53,405	45.9	
Disability status							
Yes	396	1.1	1,076	1.3	1,472	1.3	< 0.001
No	34,020	93.5	75,220	94.1	109,240	93.9	
Missing	1,969	5.4	3,606	4.5	5,575	4.8	
Pregnancy status							
Yes	n.a	n.a	11,207	14.0	n.a	n.a	n.a
No	n.a	n.a	54,208	67.8	n.a	n.a	
Not specified	n.a	n.a	14,487	18.1	n.a	n.a	

*n* number of patients, % percentage

Among all victims, physical abuse (*n* = 39,155; 33.7%) was the most common form of violence, followed by mental abuse (*n* = 38,759; 33.3%) and sexual abuse (*n* = 38,373; 33.0%) (Table 2). Among males, 73.5% (*n* = 26,743) were affected by physical abuse, whereas among females, 44.4% (*n* = 38,373) experienced sexual abuse. Family violence accounted for 57.1% (*n* = 66,407) of all IPV cases, compared to 42.9% (*n* = 49,880) for non-family violence. Gender differences were observed in family versus non-family violence, with 30.9% of males (*n* = 11,251) and 69.0% of females (*n* = 55,156) experiencing violence within the family context, respectively. Most IPV incidents occurred in residential locations (*n* = 71,934; 62.0%), affecting 73.7% (*n* = 58,750) females and 36.3% (*n* = 13,184) males. Recurrent violence was noted in 18,454 cases (18.4%), affecting 3,561 males (11.8%) and 14,893 females (21.2%). The majority of IPV victims (*n* = 109,397; 94.1%) did not receive prehospital medical attention. Medical services included emergency services (*n* = 45,478; 39.1%) for 25,515 males (70.1%) and 19,963 females (25.0%), as well as specialized violence care services (*n* = 50,779; 43.7%) for 6,362 males (17.5%) and 44,417 females (55.6%). Psychologists (*n* = 18,650; 51.0%) treated 1,786 males (23.3%) and 16,864 females (58.4%), while physicians (*n* = 14,176; 38.8%) attended to 5,217 males (68.0%) and 8,959 females (31.0%). Following treatment, most victims returned home (*n* = 26,131; 71.5%), including 5,540 males (72.2%) and 20,591 females (71.3%). For male victims, IPV medical records were highest in 2016 (*n* = 5,628; 15.5%), and for female victims, the highest number was recorded in 2022 (*n* = 14,899; 18.6%). Family violence accounted for 65.2% (*n* = 1,968) of males and 75.3% (*n* = 3,164) under 5 years old, 60.2% (*n* = 3,130) of males and 72.9% (*n* = 5,118) aged 5–9 years, 37.6% (*n* = 3,433) of males and 63.2% (*n* = 15,855) aged 10–14 years, and 14.3% (*n* = 2,720) of males and 71.2% (*n* = 31,019) aged 15–17 years (Supplementary Table 2–5).

**Table 2:** Gender-based breakdown of clinical characteristics and management of IPV victims (*n* = 116,287)

	**Male** **36,385 (31.3%)**		**Female** **79,902 (68.7%)**		**Total** **116,287 (100.0%)**		***p*-value**
	n	**%**	n	**%**	n	**%**	
Type of injury							
Physical abuse	26,743	73.5	12,412	15.5	39,155	33.7	< 0.001
Sexual abuse	3,026	8.3	35,347	44.2	38,373	33.0	
Mental abuse	6,616	18.2	32,143	40.2	38,759	33.3	
Type of violence							
Family violence	11,251	30.9	55,156	69.0	66,407	57.1	< 0.001
Non-family violence	25,134	69.1	24,746	31.0	49,880	42.9	
Location of violent event							< 0.001
Residential Locations	13,184	36.3	58,750	73.7	71,934	62.0	
School	2,890	8.0	2,159	2.7	5,049	4.4	
Recreational Locations	1,249	3.4	669	0.8	1,918	1.7	
Transportation	13,937	38.4	7,162	9.0	21,099	18.2	
Commercial location	343	0.9	591	0.7	934	0.8	
Workplace	349	1.0	357	0.4	706	0.6	
Unspecified	4,318	11.9	10,045	12.6	14,363	12.4	
Festive day							< 0.001
Yes	874	2.4	1,433	1.8	2,307	2.0	
No	35,171	96.7	77,953	97.6	113,124	97.3	
Missing	340	0.9	516	0.6	856	0.7	
Repeated violence							< 0.001
First time	15,624	51.8	19,751	28.2	35,375	35.3	
Repeated	3,561	11.8	14,893	21.2	18,454	18.4	
Missing	10,949	36.3	35,493	50.6	46,442	46.3	
Prehospital medical attention							< 0.001
Yes	3,169	8.7	3,675	4.6	6,844	5.9	
No	33,179	91.2	76,218	95.4	109,397	94.1	
Not specified	37	0.1	9	0.0	46	0.0	
Type of medical service provided							< 0.001
External consultation	3,151	8.7	10,689	13.4	13,840	11.9	
Hospitalization	1,093	3.0	2,895	3.6	3,988	3.4	
Emergency	25,515	70.1	19,963	25.0	45,478	39.1	
Specialized violence care services	6,362	17.5	44,417	55.6	50,779	43.7	
Other service	264	0.7	1,938	2.4	2,202	1.9	
Year							< 0.001
2015	5,215	14.3	7,108	8.9	12,323	10.6	
2016	5,628	15.5	8,375	10.5	14,003	12.0	
2017	4,919	13.5	8,231	10.3	13,150	11.3	
2018	4,710	12.9	9,829	12.3	14,539	12.5	
2019	3,759	10.3	10,096	12.6	13,855	11.9	
2020	2,910	8.0	8,701	10.9	11,611	10.0	
2021	4,171	11.5	12,663	15.8	16,834	14.5	
2022	5,073	13.9	14,899	18.6	19,972	17.2	

*n* number of patients, % percentage

**Fig. 1:**
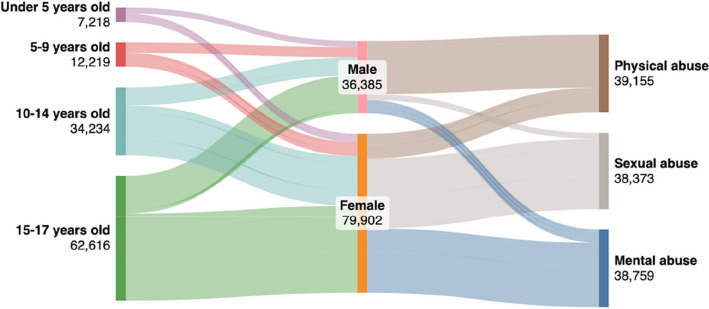
Distribution of forms of violence stratified by age category and sex (*n* = 116,287)

Physical abuse among males increased with age, affecting 47.8% (*n* = 1,441) of those under 5 years, 40.1% (*n* = 2,087) aged 5–9 years, 64.6% (*n* = 5,899) aged 10–14 years, and 91.0% (*n* = 17,316) in the 15–17 years group (Fig. 1 and Supplementary 6–9). Sexual abuse affected 17.4% (*n* = 525) of males and 44.3% (*n* = 1,862) of females under 5 years, increasing to 21.7% (*n* = 1,127) and 51.6% (*n* = 3,624) in the 5–9 years group. Among 10–14-year-olds, 10.2% (*n* = 936) of males and 52.8% (*n* = 13,244) of females were affected. In 15–17-year-olds, rates dropped to 2.3% (*n* = 438) in males but remained high at 38.1% (*n* = 16,617) in females. Mental abuse affected 34.8% (*n* = 1,051) of males and 31.4% (*n* = 1,320) of females under 5 years, increasing to 38.2% (*n* = 1,986) of males and 33.2% (*n* = 2,327) of females aged 5–9 years. Among 10–14-year-olds, 25.2% (*n* = 2,301) of males and 32.6% (*n* = 8,186) of females experienced mental abuse. In 15–17-year-olds, rates declined to 6.7% (*n* = 1,278) in males but remained high at 46.6% (*n* = 20,310) in females.

IPV cases declined during the pandemic, dropping to 11,611 cases in 2020 from 13,855 in 2019. However, cases rebounded sharply post-pandemic, rising to 16,834 in 2021 and reaching an all-time high of 19,972 in 2022.

**Fig. 2:**
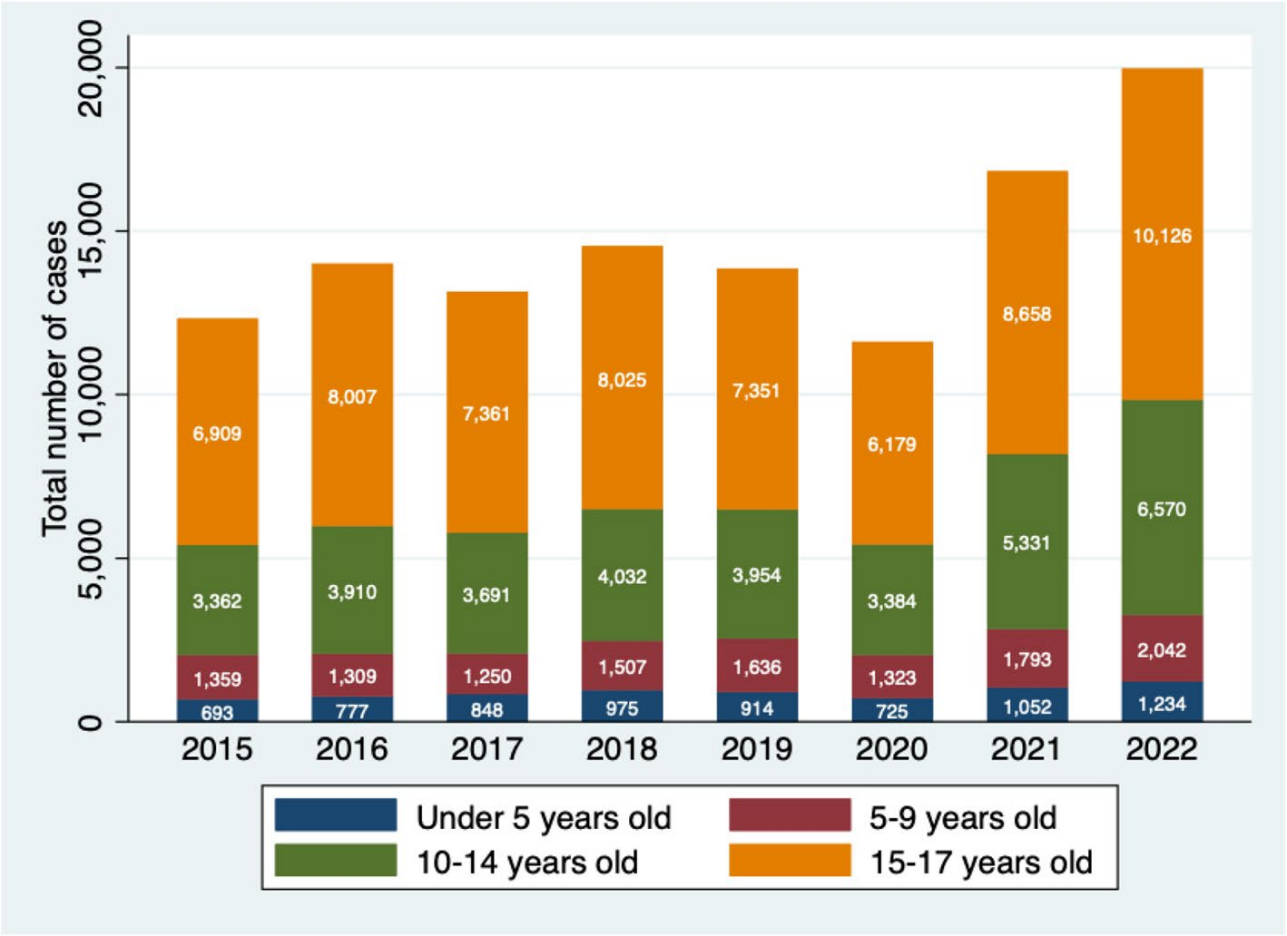
Number of IPV cases among Mexican children and adolescents (*n* = 116,287)

Among children under 5 years, numbers peaked in 2022 (*n* = 1,234; 17.1%) and were lowest in 2020 (*n* = 725; 10.0%). Similarly, for the 5–9 years age group, the highest number occurred in 2022 (*n* = 2,042; 16.7%), while the lowest was in 2017 (*n* = 1,250; 10.2%). Among 10–14-year-olds, cases were most frequent in 2022 (*n* = 6,570; 19.2%), with the lowest in 2015 (*n* = 3,362; 9.8%). The 15–17 years group followed the same pattern, with a peak in 2022 (*n* = 10,126; 16.2%) and the lowest number recorded in 2020 (*n* = 6,179; 9.9%) (Fig. 2).

**Fig. 3:**
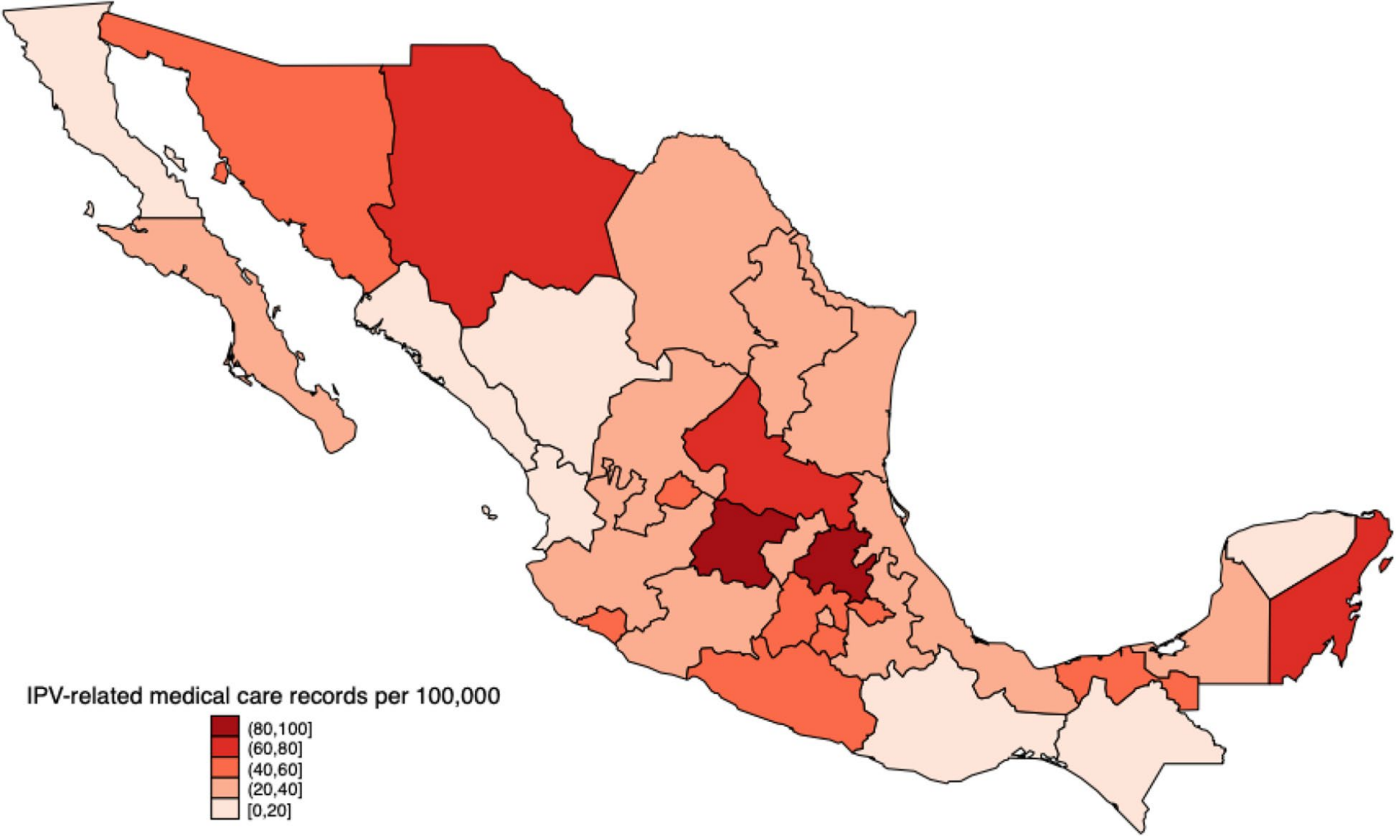
Heatmap of average number of IPV cases per 100,000 children and adolescents from 2015–2022 (*n* = 116,287)

The states with the highest average IPV cases per 100,000 children and adolescents were Chihuahua (80.0) and Guanajuato (83.8), while the lowest averages were recorded in Oaxaca (9.2) and Sinaloa (9.3) (Fig. 3 and Supplementary Table 6). The highest single-year recorded average number of cases was observed in Chihuahua (118.7 in 2022) and Hidalgo (115.9 in 2022).

The characteristics of the aggressors in IPV cases (*n* = 116,287) show that the majority were male (*n* = 76,909; 66.1%), followed by female aggressors (*n* = 9,501; 8.2%), with unspecified gender in 25.7% of cases (*n* = 29,876) (Table 3).

**Table 3:** Characteristics of the aggressors of interpersonal violence (IPV) (*n* = 116,287) in relation to their victims

	**Male victim** **36,385 (31.3%)**		**Female victim** **79,902 (68.7%)**		**Total** **116,287 (100.0%)**		***p*-value**
	**n**	**%**	**n**	**%**	n	**%**	
Sex of aggressor							
Male	16,947	46.6	59,962	75.0	76,909	66.1	< 0.001
Female	2,803	7.7	6,698	8.4	9,501	8.2	
Missing	16,634	45.7	13,242	16.6	29,876	25.7	
Age of aggressor							
Under 18 years	5,698	15.7	10,948	13.7	16,646	14.3	< 0.001
18–30 years	6,808	18.7	30,180	37.8	36,988	31.8	
31–50 years	5,282	14.5	19,115	23.9	24,397	21.0	
51–70 years	620	1.7	3,326	4.2	3,946	3.4	
More than 70 years	1,899	5.2	1,689	2.1	3,588	3.1	
Missing	16,078	44.2	14,644	18.3	30,722	26.4	
Relationship with the victim							
Biological parent	2,983	8.2	7,300	9.1	10,283	8.8	< 0.001
Spouse/partner/boyfriend	1,850	5.1	3,582	4.5	5,432	4.7	
Other relative	327	0.9	22,035	27.6	22,362	19.2	
Stepfather/mother	3,888	10.7	12,680	15.9	16,568	14.2	
Non-family acquaintance	579	1.6	4,064	5.1	4,643	4.0	
Stranger	13,410	36.9	11,758	14.7	25,168	21.6	
Other	5,658	15.6	11,126	13.9	16,784	14.4	
Not specified	7,690	21.1	7,357	9.2	15,047	12.9	

*n* number of patients, % percentage

In terms of age, the most represented group was individuals aged 18–30 years (*n* = 36,988; 31.8%), followed by those aged 31–50 years (*n* = 24,397; 21.0%), and those under 18 years (*n* = 16,646; 14.3%). Female aggressors were reported in 19.1% (*n* = 577) of cases for male victims under 5 years and 15.3% (*n* = 641) for female victims (Supplementary Table 7–9). Among 15–17-year-olds, female aggressors were involved in 2.6% (*n* = 496) for male victims and 6.6% (= 2,858) for female victims. Strangers were reported in 16.3% (*n* = 493) of cases for males and 12.7% (*n* = 535) for females under 5 years, 15.9% (*n* = 828) for males and 11.1% (*n* = 780) for females in ages 5–9, 29.1% (*n* = 2,659) for males and 16.2% (*n* = 4,064) for females in ages 10–14, and 49.5% (*n* = 9,430) for males and 14.6% (*n* = 6,379) for females in ages 15–17.

## Discussion

### Overview of IPV cases of children and adolescents in Mexico

This study provides insights into the epidemiology of IPV cases among children and adolescents in Mexico using a registry-based analysis. At the individual level, adolescent girls are disproportionately affected by mental and sexual abuse. At the relationship level, IPV predominantly occurs in family contexts, reinforcing the role of familial power dynamics. At the community level, geographic disparities in IPV rates suggest the influence of socio-economic inequalities. Finally, at the societal level, entrenched gender norms and weak law enforcement contribute to the persistence of IPV. These findings underscore the need for multi-level interventions addressing IPV at each level.

### Gender disparities among IPV victims

The analysis reveals a stark gender disparity among IPV victims, with females constituting 68.7% of the medical records. This finding aligns with previous studies in Mexico and other Latin American countries, where females are disproportionately affected by IPV [35]. A report found that 80.8% of victims of family and non-family violence in Mexico in 2022 were girls and female adolescents [36]. The higher prevalence of female victims may be attributed to societal norms and gender inequalities that perpetuate violence against women. The situation of females being victims of sexual violence more frequently than males is exacerbated by gender inequality [7]. Our study found that the majority of aggressors were male (66.1%), consistent with global IPV patterns [37]. This is supported by another study from Mexico on child sexual abuse, where the aggressor was a male in 79.7% of cases, aligning with our findings [38]. A study from Argentina also highlighted the gendered nature of IPV, noting that females were more likely to suffer from violence inflicted by male partners or relatives [35]. Moreover, existing literature outlines patterns regarding victims’ perpetrators, including partners for women and strangers for men [39]. Mexico has recognized the issue of violence, particularly gender-based violence, as a national priority and has implemented policies aimed at protecting children and adolescents from IPV. The National System for the Comprehensive Protection of Children and Adolescents (SIPINNA) serves as Mexico’s highest political decision-making body dedicated to safeguarding and promoting the rights of minors [40, 41]. Established in 2014, SIPINNA operates as a coordinated network of policies, institutions, and actors across all levels of government. Through its Executive Secretariat, it plays a crucial role in implementing the General Law for the Protection of the Rights of Children and Adolescents, aiming to strengthen multi-sectoral responses to IPV [42].

To eliminate the causes of violence against girls and female adolescents, tailored, cross-sectoral approaches addressing sociocultural attitudes are essential, as demonstrated by the"100-Day Challenge"in Chihuahua, which achieved a 193% increase in resolved violence cases in Ciudad Juarez [43].

### Types of violence

Physical abuse was most prevalent (33.7%) and it predominantly affected males (73.5%). This aligns with evidence from a study conducted in Argentina, which reported a higher likelihood of physical abuse among male adolescents compared to their female counterparts [35]. Research conducted in the United States has documented higher instances of physical abuse among male children and adolescents, further corroborating our findings [44]. This situation can partly be attributed to the use of corporal punishment by caretakers or teachers commonly observed among males [45, 46], and fights between boys or beatings by their peers [47]. Community interventions such as the"Familias en Acción"project in Colombia use community-based participatory research to actively involve local members in violence prevention efforts, leading to sustained benefits [48]. In states with high IPV rates, implementing these culturally tailored community interventions is especially critical to empower youth, mobilize local communities, and foster sustainable reductions in IPV.

Mental abuse was the most prevalent form accounting for 33.3% of documented IPVs, affecting 40.2% of female victims. This finding aligns with data from a general population survey by *INEGI*, which states that 51.6% of women aged 15 years and older have experienced mental violence throughout their life [49]. It has been shown that mental abuse can have more debilitating long-term effects than physical abuse, emphasizing the need for targeted mental health interventions [50]. Additionally, mental aggression has been consistently identified as a predictor of future physical violence in relationships [51]. To address this issue, it is crucial to implement preventive education programs and ensure accessible mental health services for victims of mental abuse. School-based interventions have been proven to decrease the detrimental effects of mental abuse [52]. Culturally tailored school interventions like the adaptation of"El Joven Noble"for children teach conflict resolution, promote non-violent self-efficacy, and challenge gender norms [53],

Sexual abuse particularly affected girls aged 5–9 (51.6%) and 10–14 (52.8%), indicating a high prevalence of sexual exploitation and coercion among young girls. The national data from *INEGI* reported similar results whereby 49.7% of females aged 15 and above have experienced sexual violence [49]. Notably, another study from Mexico found that 77.7% of surveyed women suffered at least one episode of sexual abuse during their childhood [38], while in Mexico City, a study with students from a public school found that 45.5% of female students were involved in a “sexual situation” against their will [54]. Moreover, a study by Benjet et. al. focusing on chronic childhood adversities found that 3.0% of female students regularly experienced sexual abuse [55]. Since most IPV cases are perpetrated by males older than victims and occur at home or in residential areas, female adolescents are often exploited by people that they trust, including their family members. This shows the necessity for protective environment and social norms and for girls’ empowerment and support measures. These measures include sexual education on skills to prevent sexual violence, consent awareness, how to alert when in danger, and self-defense training [56]. The"Paint Your Stripe ASI (CSA)"program, a research project by the National Institute of Public Health of Mexico, has shown the effectiveness of awareness-raising workshops in laying the groundwork for preventing child sexual abuse [57]. The continuation of policy changes initiated by the previous administration, including the restructuring or discontinuation of certain health programs, has increased the vulnerability of victims of violence [58].

### Location of IPV

In our study, most IPV incidents among children and adolescents in Mexico occurred in residential areas (at home), particularly among females. The high concentration of IPV cases in residential settings compared to other areas in our study can likely be attributed, at least in part, to the COVID-19 pandemic and the resulting lockdown measures, which increased the time children and adolescents were exposed to their aggressors, as demonstrated in other studies [59–61]. A study in the USA highlights that family-level interventions, such as the Fathers for Change (F4 C) program, which focus on improving emotion regulation and reflective functioning in fathers, are associated with lower rates of repeat maltreatment [62]. The"Cara y Corazón” involves Mexican–American families in violence prevention through monthly retreats and activities that foster non-violent behaviors and strengthen family bonds [63].

### Number of IPV cases during the pandemic

The number of documented IPV cases showed a decline during the pandemic years with a sharp increase after the pandemic. This finding contrasts with the majority of COVID-19-related literature, which mainly reported an increase in IPV cases during the pandemic: Most of these studies attributed this rise to heightened stress, economic hardship, and confinement measures that exacerbated tensions within households [64–66]. National data from Brazil showed an 18% increase in violence-related complaints in 2020 [67]. Other studies reported a decline in IPV cases during COVID-19 [68, 69], attributing this decrease to underreporting of cases and documentation problems during lockdown. A study from Colorado found a 31% decline in child maltreatment (including IPV) during the COVID-19 pandemic, largely attributed to reduced reporting due to limited interactions with mandatory reporters such as teachers and healthcare providers during stay-at-home orders and school closures [70]. Similarly, another study from the USA reported fewer cases of child physical abuse during the pandemic, likely due to underreporting by mandatory reporter [71]. A study by Mohajed et al. further emphasized the decrease in institutional responses to violence against children and adolescents, noting that limited access to community services and mandatory reporters led to underreported cases despite an increase in severe incident [72]. Further, there are several reasons for why parents fail to report child abuse and violence. These reasons include fear, denial, lack of resources, and cultural factors [73]. A comprehensive approach is required to address this issue, including awareness raising, support, and building trust. Overall, while IPV cases largely increased during the pandemic, studies involving children frequently reported decreases due to significant underreporting issues, underscoring the critical role of mandatory reporters in identifying and addressing violence. This discrepancy highlights the complex dynamics of IPV during crises and the urgent need for robust systems to monitor and support vulnerable populations.

### Geographic variations in IPV rates

The geographical analysis revealed variations in the average number of IPV cases Mexican states Guanajuato (83.8) and Chihuahua (80.0) exhibited the highest average IPV cases per 100,000 children and adolescents. The persistent high average number of IPV cases in these states suggest underlying socio-economic, cultural, and possibly systemic factors contributing to IPV prevalence. This aligns with findings from a 2019 study, which reported that Guanajuato had the highest number of homicides in the country [74]. Chihuahua has historically had one of the highest murder and crime rates in Mexico [75]. These outcomes reflect the role of socio-economic disparities and entrenched cultural norms in higher average numbers of IPV cases Furthermore, research from Mexico indicates that states with higher levels of economic inequality and lower educational attainment tend to have higher rates of IPV [37]. To address interpersonal violence effectively, it is imperative to train primary care providers to screen, treat, and refer affected patients; strengthen community health centers by equipping them with necessary resources to manage violence cases; and develop culturally responsive services in low-resource settings that integrate mental health care with violence intervention [76, 77]. Additionally, the Spotlight Initiative led by UNICEF and SIPINNA aim to establish safe spaces in states like Chihuahua, promoting life skills and violence prevention among children and adolescents [78].

### Study strengths

This study had several strengths. It utilized nationwide data with a robust sample size, enhancing the generalizability of the findings to the broader population of Mexico. The comprehensive nature of the data allowed for a detailed analysis of IPV patterns and trends across various demographics and regions, providing a clear picture of the scope and nature of the issue. This level of detail supports the development of targeted interventions and policies tailored to the specific needs of different groups, ensuring that prevention and support efforts are as effective as possible.

### Study limitations

Nonetheless, this study is not without limitations. The use of registry-based data may lead to underreporting of IPV, as not all incidents result in medical records at health facilities. This is particularly true for mental and sexual abuse, which may be less likely to be documented compared to physical abuse. Missing values and reporting issues, especially during the pandemic, may further affect the completeness and accuracy of the medical records. Additionally, this study is limited to data from public health facilities, potentially excluding medical records from private clinics or incidents where care was not sought at all. Furthermore, the data includes only patients who sought hospital care, meaning the true extent of IPV is likely much larger. The *Lesiones* database used in this study represents only a portion of the IPV cases, and its scope does not fully capture all cases. Finally, the cross-sectional nature of the data limits our ability to establish causality or long-term trends beyond the study period.

## Conclusion

This study described the epidemiology of IPV cases among children and adolescents in Mexico. Two-thirds of cases were classified as family violence, emphasizing the need for targeted domestic interventions. Framed within the Social-Ecological Model (SEM), these findings highlight the necessity of multi-sectoral strategies, including individual empowerment programs, family-focused (relationship level) interventions, community-based reporting mechanisms, and strengthened legal enforcement to challenge societal norms perpetuating violence. In line with international human rights law, governments must implement protective policies, ensure access to justice, and provide gender-sensitive training for professionals [18]. Future research should continue to explore the underlying causes of IPV against children and adolescents and evaluate the effectiveness of interventions to inform evidence-based policies. These should include comprehensive legal frameworks that condemn violence, culturally tailored interventions for families, schools, and communities, increased professional training for healthcare, judicial, and law enforcement sectors, and improved access to justice and support services for victims.

## Supplementary Information

Supplementary Material 1.

## Data Availability

The Lesiones database, as stated and cited in the methods, which provides comprehensive data on injuries in Mexico, is publicly accessible at https://www.datos.gob.mx/busca/dataset/lesiones.
