# Supplementary material for: Epidemiology of interpersonal violence among Mexican children and adolescents: a national analysis of injury data from public hospitals from 2015 to 2022
Source: BMC Public Health. 2025 May 9;25:1712. doi: 10.1186/s12889-025-22990-z (PMC12063275; doi:10.1186/s12889-025-22990-z)
Supplement: Supplementary file 1 — Supplementary Material 1. [file 12889_2025_22990_MOESM1_ESM.docx]

# Supplementary

**Supplementary Table 1:** Categorization of interpersonal violence (IPV) with corresponding ICD-10 codes

| **Physical Abuse:** | T07X, T887, T809, S101, S207, S407, S497, S498, S507, S707, S807, S907, S997, T002, S597, S598, S508, S997, S999, S599, S899, T139, S199, S499, S299, T099, T119, S699, T71X, T33*, T8*, S902, T34*, T35*, S709, S808, S908, S911, S912, S998, S718, S200, S24*, S25*, S26*, S27*, S28*, S35*, S36*, S37*, S38*, S47*, S48*, S44*, S45*, S46*, S54*, S55*, S56*, S57*, S64*, S65*, S66*, S67*, S68*, S74*, S75*, S76*, S77*, S78*, S79*, S84*, S85*, S86*, S87*, S88*, S89*, S94*, S95*, S96*, S97*, S98*, T04*, T05*, T06*, T09*, T14*, S301, S900, S500, S302, S051, S001, S601, S600, S700, S800, S300, S903, S501, S801, S602, S400, S701, S202, S100, S901, S431, S220, S224, S229, S930, S131, S531, S631, S030, S831, S132, S031, S430, S630, T143, T092, T030, T033, T032, S920, S420, S729, S929, S529, S826, S526, S424, S525, S829, S923, S620, S720, S129, S328, S628, S621, S626, S623, S228, S528, S428, S122, S922, S925, S22*, S23*, S32*, S33*, S34*, S42*, S43*, S62*, S63*, S72*, S73*, S82*, S83*, S92*, S93*, T02*, T03*, S530, S532, S820, S828, S223, S421, S723, S423, S523, S822, S522, S524, T142, S422, S521, S530, S821, S520, T026, S427, S727, S627, S527, S827, S624, T029, T10X, T112, T116, T12X, T132, S018, S019, S009, S099 |
| --- | --- |
| **Sexual Abuse:** | T742, S380 |
| **Mental Abuse** | F*, T743, T749, T748, T741, T740 |

**Supplementary Table 2:** Gender-based breakdown of clinical characteristics and management of IPV victims (Under 5 years old) (n=7,218)

|  |  | **Male**  **3,017 (41.8%)** | **Female**  **4,201**  **(58.2%)** | **Total**  **7,218 (100.0%)** | **p-value** |
| --- | --- | --- | --- | --- | --- |
|  |  | **n (%)** | **n (%)** | **n (%)** |  |
| **Type of injury** | Physical abuse | 1,441 (47.8%) | 1,019 (24.3%) | 2,460 (34.1%) | <0.001 |
|  | Sexual abuse | 525 (17.4%) | 1,862 (44.3%) | 2,387 (33.1%) |  |
|  | Mental abuse | 1,051 (34.8%) | 1,320 (31.4%) | 2,371 (32.8%) |  |
| **Type of violence** | Family violence | 1,968 (65.2%) | 3,164 (75.3%) | 5,132 (71.1%) | <0.001 |
|  | Non-family violence | 1,049 (34.8%) | 1,037 (24.7%) | 2,086 (28.9%) |  |
| **Location of violent event** | Residential Locations | 2,097 (69.7%) | 3,322 (79.3%) | 5,419 (75.3%) | <0.001 |
|  | School | 143 ( 4.8%) | 158 ( 3.8%) | 301 ( 4.2%) |  |
|  | Recreational Locations | 26 ( 0.9%) | 16 ( 0.4%) | 42 ( 0.6%) |  |
|  | Transportation | 384 (12.8%) | 207 ( 4.9%) | 591 ( 8.2%) |  |
|  | Commercial location | 21 ( 0.7%) | 20 ( 0.5%) | 41 ( 0.6%) |  |
|  | Workplace | 10 ( 0.3%) | 11 ( 0.3%) | 21 ( 0.3%) |  |
|  | Unspecified | 328 (10.9%) | 457 (10.9%) | 785 (10.9%) |  |
| **Festive day** | Yes | 62 ( 2.1%) | 72 ( 1.7%) | 134 ( 1.9%) | 0.085 |
|  | No | 2,914 (96.6%) | 4,092 (97.4%) | 7,006 (97.1%) |  |
|  | Missing | 41 ( 1.4%) | 37 ( 0.9%) | 78 ( 1.1%) |  |
| **Repeated violence** | First time | 1,064 (41.4%) | 1,324 (35.3%) | 2,388 (37.8%) | <0.001 |
|  | Repeated | 356 (13.8%) | 566 (15.1%) | 922 (14.6%) |  |
|  | Missing | 1,152 (44.8%) | 1,857 (49.6%) | 3,009 (47.6%) |  |
| **Prehospital medical attention** | Yes | 310 (10.3%) | 323 ( 7.7%) | 633 ( 8.8%) | <0.001 |
|  | No | 2,702 (89.6%) | 3,877 (92.3%) | 6,579 (91.1%) |  |
|  | Not specified | 5 ( 0.2%) | 1 ( 0.0%) | 6 ( 0.1%) |  |
| **Referral institution** | Medical facility | 232 ( 7.7%) | 320 ( 7.6%) | 552 ( 7.6%) | <0.001 |
|  | Non-medical governmental institution | 494 (16.4%) | 1,025 (24.4%) | 1,519 (21.0%) |  |
|  | Non-governmental organization | 1,195 (39.6%) | 1,292 (30.8%) | 2,487 (34.5%) |  |
|  | Self-referred | 749 (24.8%) | 1,141 (27.2%) | 1,890 (26.2%) |  |
|  | Not specified | 347 (11.5%) | 423 (10.1%) | 770 (10.7%) |  |
| **Type of medical service provided** | External consultation | 339 (11.2%) | 530 (12.6%) | 869 (12.0%) | <0.001 |
|  | Hospitalization | 98 ( 3.2%) | 117 ( 2.8%) | 215 ( 3.0%) |  |
|  | Emergency | 1,794 (59.5%) | 1,859 (44.3%) | 3,653 (50.6%) |  |
|  | Specialized violence care services | 718 (23.8%) | 1,569 (37.3%) | 2,287 (31.7%) |  |
|  | Other service | 68 ( 2.3%) | 126 ( 3.0%) | 194 ( 2.7%) |  |
| **Type of care provider** | Physician | 1,879 (62.3%) | 2,367 (56.3%) | 4,246 (58.8%) | <0.001 |
|  | Psychologist | 534 (17.7%) | 1,114 (26.5%) | 1,648 (22.8%) |  |
|  | Social worker | 454 (15.0%) | 503 (12.0%) | 957 (13.3%) |  |
|  | Not specified | 150 ( 5.0%) | 217 ( 5.2%) | 367 ( 5.1%) |  |
| **Post-treatment destination** | Home | 1,679 (55.7%) | 2,160 (51.4%) | 3,839 (53.2%) | <0.001 |
|  | Transfer to another medical unit | 100 ( 3.3%) | 119 ( 2.8%) | 219 ( 3.0%) |  |
|  | Violence response service | 324 (10.7%) | 717 (17.1%) | 1,041 (14.4%) |  |
|  | External consultation | 67 ( 2.2%) | 98 ( 2.3%) | 165 ( 2.3%) |  |
|  | Deceased | 13 ( 0.4%) | 13 ( 0.3%) | 26 ( 0.4%) |  |
|  | Subsequent hospitalization | 383 (12.7%) | 374 ( 8.9%) | 757 (10.5%) |  |
|  | Other | 451 (14.9%) | 720 (17.1%) | 1,171 (16.2%) |  |
| **Year** | 2015 | 330 (10.9%) | 363 ( 8.6%) | 693 ( 9.6%) | <0.001 |
|  | 2016 | 389 (12.9%) | 388 ( 9.2%) | 777 (10.8%) |  |
|  | 2017 | 383 (12.7%) | 465 (11.1%) | 848 (11.7%) |  |
|  | 2018 | 423 (14.0%) | 552 (13.1%) | 975 (13.5%) |  |
|  | 2019 | 350 (11.6%) | 564 (13.4%) | 914 (12.7%) |  |
|  | 2020 | 266 ( 8.8%) | 459 (10.9%) | 725 (10.0%) |  |
|  | 2021 | 403 (13.4%) | 649 (15.4%) | 1,052 (14.6%) |  |
|  | 2022 | 473 (15.7%) | 761 (18.1%) | 1,234 (17.1%) |  |

n: number of patients; %: percentage

**Supplementary Table 3:** Gender-based breakdown of clinical characteristics and management of IPV victims (5-9 years old) (n=12,219)

|  |  | **Male**  **5,200 (42.6%)** | **Female**  **7,019 (57.4%)** | **Total**  **12,219 (100.0%)** | **p-value** |
| --- | --- | --- | --- | --- | --- |
|  |  | **n (%)** | **n (%)** | **n (%)** |  |
| **Type of injury** | Physical abuse | 2,087 (40.1%) | 1,068 (15.2%) | 3,155 (25.8%) | <0.001 |
|  | Sexual abuse | 1,127 (21.7%) | 3,624 (51.6%) | 4,751 (38.9%) |  |
|  | Mental abuse | 1,986 (38.2%) | 2,327 (33.2%) | 4,313 (35.3%) |  |
| **Type of violence** | Family violence | 3,130 (60.2%) | 5,118 (72.9%) | 8,248 (67.5%) | <0.001 |
|  | Non-family violence | 2,070 (39.8%) | 1,901 (27.1%) | 3,971 (32.5%) |  |
| **Location of violent event** | Residential Locations | 3,415 (65.7%) | 5,675 (80.9%) | 9,090 (74.5%) | <0.001 |
|  | School | 762 (14.7%) | 378 ( 5.4%) | 1,140 ( 9.3%) |  |
|  | Recreational Locations | 96 ( 1.8%) | 35 ( 0.5%) | 131 ( 1.1%) |  |
|  | Transportation | 485 ( 9.3%) | 273 ( 3.9%) | 758 ( 6.2%) |  |
|  | Commercial location | 24 ( 0.5%) | 46 ( 0.7%) | 70 ( 0.6%) |  |
|  | Workplace | 15 ( 0.3%) | 8 ( 0.1%) | 23 ( 0.2%) |  |
|  | Unspecified | 397 ( 7.6%) | 596 ( 8.5%) | 993 ( 8.1%) |  |
| **Festive day** | Yes | 89 ( 1.7%) | 118 ( 1.7%) | 207 ( 1.7%) | 0.007 |
|  | No | 5,076 (97.6%) | 6,881 (98.0%) | 11,957 (97.9%) |  |
|  | Missing | 35 ( 0.7%) | 20 ( 0.3%) | 55 ( 0.5%) |  |
| **Repeated violence** | First time | 1,550 (34.9%) | 1,644 (26.1%) | 3,194 (29.7%) | <0.001 |
|  | Repeated | 1,118 (25.1%) | 1,469 (23.3%) | 2,587 (24.1%) |  |
|  | Missing | 1,779 (40.0%) | 3,193 (50.6%) | 4,972 (46.2%) |  |
| **Prehospital medical attention** | Yes | 299 ( 5.8%) | 387 ( 5.5%) | 686 ( 5.6%) | 0.20 |
|  | No | 4,897 (94.2%) | 6,631 (94.5%) | 11,528 (94.3%) |  |
|  | Not specified | 4 ( 0.1%) | 1 ( 0.0%) | 5 ( 0.0%) |  |
| **Referral institution** | Medical facility | 283 ( 5.4%) | 447 ( 6.4%) | 730 ( 6.0%) | <0.001 |
|  | Non-medical governmental institution | 1,214 (23.3%) | 2,533 (36.1%) | 3,747 (30.7%) |  |
|  | Non-governmental organization | 2,033 (39.1%) | 1,753 (25.0%) | 3,786 (31.0%) |  |
|  | Self-referred | 1,315 (25.3%) | 1,791 (25.5%) | 3,106 (25.4%) |  |
|  | Not specified | 355 ( 6.8%) | 495 ( 7.1%) | 850 ( 7.0%) |  |
| **Type of medical service provided** | External consultation | 615 (11.8%) | 775 (11.0%) | 1,390 (11.4%) | <0.001 |
|  | Hospitalization | 63 ( 1.2%) | 65 ( 0.9%) | 128 ( 1.0%) |  |
|  | Emergency | 2,226 (42.8%) | 2,064 (29.4%) | 4,290 (35.1%) |  |
|  | Specialized violence care services | 2,243 (43.1%) | 3,936 (56.1%) | 6,179 (50.6%) |  |
|  | Other service | 53 ( 1.0%) | 179 ( 2.6%) | 232 ( 1.9%) |  |
| **Type of care provider** | Physician | 2,704 (52.0%) | 3,172 (45.2%) | 5,876 (48.1%) | <0.001 |
|  | Psychologist | 1,465 (28.2%) | 2,639 (37.6%) | 4,104 (33.6%) |  |
|  | Social worker | 854 (16.4%) | 954 (13.6%) | 1,808 (14.8%) |  |
|  | Not specified | 177 ( 3.4%) | 254 ( 3.6%) | 431 ( 3.5%) |  |
| **Post-treatment destination** | Home | 3,723 (71.6%) | 4,298 (61.2%) | 8,021 (65.6%) | <0.001 |
|  | Transfer to another medical unit | 116 ( 2.2%) | 150 ( 2.1%) | 266 ( 2.2%) |  |
|  | Violence response service | 551 (10.6%) | 1,179 (16.8%) | 1,730 (14.2%) |  |
|  | External consultation | 126 ( 2.4%) | 175 ( 2.5%) | 301 ( 2.5%) |  |
|  | Deceased | 9 ( 0.2%) | 5 ( 0.1%) | 14 ( 0.1%) |  |
|  | Subsequent hospitalization | 222 ( 4.3%) | 275 ( 3.9%) | 497 ( 4.1%) |  |
|  | Other | 452 ( 8.7%) | 937 (13.3%) | 1,389 (11.4%) |  |
|  | Not specified | 1 ( 0.0%) | 0 ( 0.0%) | 1 ( 0.0%) |  |
| **Year** | 2015 | 668 (12.8%) | 691 ( 9.8%) | 1,359 (11.1%) | <0.001 |
|  | 2016 | 681 (13.1%) | 628 ( 8.9%) | 1,309 (10.7%) |  |
|  | 2017 | 626 (12.0%) | 624 ( 8.9%) | 1,250 (10.2%) |  |
|  | 2018 | 707 (13.6%) | 800 (11.4%) | 1,507 (12.3%) |  |
|  | 2019 | 654 (12.6%) | 982 (14.0%) | 1,636 (13.4%) |  |
|  | 2020 | 469 ( 9.0%) | 854 (12.2%) | 1,323 (10.8%) |  |
|  | 2021 | 657 (12.6%) | 1,136 (16.2%) | 1,793 (14.7%) |  |
|  | 2022 | 738 (14.2%) | 1,304 (18.6%) | 2,042 (16.7%) |  |

n: number of patients; %: percentage

**Supplementary Table 4:** Gender-based breakdown of clinical characteristics and management of IPV victims (10-14 years old) (n=34,234)

|  |  | **Male**  **9,136 (26.7%)** | **Female**  **25,098 (73.3%)** | **Total**  **34,234 (100.0%)** | **p-value** |
| --- | --- | --- | --- | --- | --- |
|  |  | **n (%)** | **n (%)** | **n (%)** |  |
| **Type of injury** | Physical abuse | 5,899 (64.6%) | 3,668 (14.6%) | 9,567 (27.9%) | <0.001 |
|  | Sexual abuse | 936 (10.2%) | 13,244 (52.8%) | 14,180 (41.4%) |  |
|  | Mental abuse | 2,301 (25.2%) | 8,186 (32.6%) | 10,487 (30.6%) |  |
| **Type of violence** | Family violence | 3,433 (37.6%) | 15,855 (63.2%) | 19,288 (56.3%) | <0.001 |
|  | Non-family violence | 5,703 (62.4%) | 9,243 (36.8%) | 14,946 (43.7%) |  |
| **Location of violent event** | Residential Locations | 3,885 (42.6%) | 17,843 (71.2%) | 21,728 (63.6%) | <0.001 |
|  | School | 1,508 (16.5%) | 987 ( 3.9%) | 2,495 ( 7.3%) |  |
|  | Recreational Locations | 333 ( 3.7%) | 259 ( 1.0%) | 592 ( 1.7%) |  |
|  | Transportation | 2,478 (27.2%) | 2,228 ( 8.9%) | 4,706 (13.8%) |  |
|  | Commercial location | 66 ( 0.7%) | 209 ( 0.8%) | 275 ( 0.8%) |  |
|  | Workplace | 60 ( 0.7%) | 70 ( 0.3%) | 130 ( 0.4%) |  |
|  | Unspecified | 782 ( 8.6%) | 3,449 (13.8%) | 4,231 (12.4%) |  |
| **Festive day** | Yes | 195 ( 2.1%) | 492 ( 2.0%) | 687 ( 2.0%) | <0.001 |
|  | No | 8,850 (96.9%) | 24,501 (97.6%) | 33,351 (97.4%) |  |
|  | Missing | 91 ( 1.0%) | 105 ( 0.4%) | 196 ( 0.6%) |  |
| **Repeated violence** | First time | 3,545 (46.7%) | 6,136 (27.6%) | 9,681 (32.5%) | <0.001 |
|  | Repeated | 1,243 (16.4%) | 4,129 (18.6%) | 5,372 (18.0%) |  |
|  | Missing | 2,804 (36.9%) | 11,942 (53.8%) | 14,746 (49.5%) |  |
| **Prehospital medical attention** | Yes | 689 ( 7.5%) | 1,219 ( 4.9%) | 1,908 ( 5.6%) | <0.001 |
|  | No | 8,433 (92.3%) | 23,873 (95.1%) | 32,306 (94.4%) |  |
|  | Not specified | 14 ( 0.2%) | 6 ( 0.0%) | 20 ( 0.1%) |  |
| **Referral institution** | Medical facility | 462 ( 5.1%) | 1,873 ( 7.5%) | 2,335 ( 6.8%) | <0.001 |
|  | Non-medical governmental institution | 1,380 (15.1%) | 8,784 (35.0%) | 10,164 (29.7%) |  |
|  | Non-governmental organization | 4,390 (48.1%) | 5,833 (23.2%) | 10,223 (29.9%) |  |
|  | Self-referred | 2,372 (26.0%) | 6,585 (26.2%) | 8,957 (26.2%) |  |
|  | Not specified | 532 ( 5.8%) | 2,023 ( 8.1%) | 2,555 ( 7.5%) |  |
| **Type of medical service provided** | External consultation | 1,074 (11.8%) | 3,014 (12.0%) | 4,088 (11.9%) | <0.001 |
|  | Hospitalization | 238 ( 2.6%) | 502 ( 2.0%) | 740 ( 2.2%) |  |
|  | Emergency | 5,507 (60.3%) | 6,648 (26.5%) | 12,155 (35.5%) |  |
|  | Specialized violence care services | 2,242 (24.5%) | 14,308 (57.0%) | 16,550 (48.3%) |  |
|  | Other service | 75 ( 0.8%) | 626 ( 2.5%) | 701 ( 2.0%) |  |
| **Type of care provider** | Physician | 6,435 (70.4%) | 10,383 (41.4%) | 16,818 (49.1%) | <0.001 |
|  | Psychologist | 1,698 (18.6%) | 10,839 (43.2%) | 12,537 (36.6%) |  |
|  | Social worker | 735 ( 8.0%) | 2,900 (11.6%) | 3,635 (10.6%) |  |
|  | Not specified | 268 ( 2.9%) | 976 ( 3.9%) | 1,244 ( 3.6%) |  |
| **Post-treatment destination** | Home | 6,592 (72.2%) | 15,531 (61.9%) | 22,123 (64.6%) | <0.001 |
|  | Transfer to another medical unit | 354 ( 3.9%) | 381 ( 1.5%) | 735 ( 2.1%) |  |
|  | Violence response service | 682 ( 7.5%) | 5,101 (20.3%) | 5,783 (16.9%) |  |
|  | External consultation | 355 ( 3.9%) | 785 ( 3.1%) | 1,140 ( 3.3%) |  |
|  | Deceased | 16 ( 0.2%) | 9 ( 0.0%) | 25 ( 0.1%) |  |
|  | Subsequent hospitalization | 544 ( 6.0%) | 740 ( 2.9%) | 1,284 ( 3.8%) |  |
|  | Other | 593 ( 6.5%) | 2,550 (10.2%) | 3,143 ( 9.2%) |  |
|  | Not specified | 0 ( 0.0%) | 1 ( 0.0%) | 1 ( 0.0%) |  |
| **Year** | 2015 | 1,257 (13.8%) | 2,105 ( 8.4%) | 3,362 ( 9.8%) | <0.001 |
|  | 2016 | 1,380 (15.1%) | 2,530 (10.1%) | 3,910 (11.4%) |  |
|  | 2017 | 1,239 (13.6%) | 2,452 ( 9.8%) | 3,691 (10.8%) |  |
|  | 2018 | 1,190 (13.0%) | 2,842 (11.3%) | 4,032 (11.8%) |  |
|  | 2019 | 988 (10.8%) | 2,966 (11.8%) | 3,954 (11.5%) |  |
|  | 2020 | 707 ( 7.7%) | 2,677 (10.7%) | 3,384 ( 9.9%) |  |
|  | 2021 | 967 (10.6%) | 4,364 (17.4%) | 5,331 (15.6%) |  |
|  | 2022 | 1,408 (15.4%) | 5,162 (20.6%) | 6,570 (19.2%) |  |

n: number of patients; %: percentage

**Supplementary Table 5:** Gender-based breakdown of clinical characteristics and management of IPV victims (15-17 years old) (n=62,616)

|  |  | **Male**  **19,032 (30.4%)** | **Female**  **43,584**  **(69.6%)** | **Total**  **62,616**  **(100.0%)** | **p-value** |
| --- | --- | --- | --- | --- | --- |
|  |  | **n (%)** | **n (%)** | **n (%)** |  |
| **Type of injury** | Physical abuse | 17,316 (91.0%) | 6,657 (15.3%) | 23,973 (38.3%) | <0.001 |
|  | Sexual abuse | 438 ( 2.3%) | 16,617 (38.1%) | 17,055 (27.2%) |  |
|  | Mental abuse | 1,278 ( 6.7%) | 20,310 (46.6%) | 21,588 (34.5%) |  |
| **Type of violence** | Family violence | 2,720 (14.3%) | 31,019 (71.2%) | 33,739 (53.9%) | <0.001 |
|  | Non-family violence | 16,312 (85.7%) | 12,565 (28.8%) | 28,877 (46.1%) |  |
| **Location of violent event** | Residential Locations | 3,787 (20.0%) | 31,910 (73.4%) | 35,697 (57.2%) | <0.001 |
|  | School | 477 ( 2.5%) | 636 ( 1.5%) | 1,113 ( 1.8%) |  |
|  | Recreational Locations | 794 ( 4.2%) | 359 ( 0.8%) | 1,153 ( 1.8%) |  |
|  | Transportation | 10,590 (55.9%) | 4,454 (10.2%) | 15,044 (24.1%) |  |
|  | Commercial location | 232 ( 1.2%) | 316 ( 0.7%) | 548 ( 0.9%) |  |
|  | Workplace | 264 ( 1.4%) | 268 ( 0.6%) | 532 ( 0.9%) |  |
|  | Unspecified | 2,811 (14.8%) | 5,543 (12.7%) | 8,354 (13.4%) |  |
| **Festive day** | Yes | 528 ( 2.8%) | 751 ( 1.7%) | 1,279 ( 2.0%) | <0.001 |
|  | No | 18,331 (96.3%) | 42,479 (97.5%) | 60,810 (97.1%) |  |
|  | Missing | 173 ( 0.9%) | 354 ( 0.8%) | 527 ( 0.8%) |  |
| **Repeated violence** | First time | 9,465 (61.0%) | 10,647 (28.1%) | 20,112 (37.7%) | <0.001 |
|  | Repeated | 844 ( 5.4%) | 8,729 (23.0%) | 9,573 (17.9%) |  |
|  | Missing | 5,214 (33.6%) | 18,501 (48.8%) | 23,715 (44.4%) |  |
| **Prehospital medical attention** | Yes | 1,871 ( 9.8%) | 1,746 ( 4.0%) | 3,617 ( 5.8%) | <0.001 |
|  | No | 17,147 (90.1%) | 41,837 (96.0%) | 58,984 (94.2%) |  |
|  | Not specified | 14 ( 0.1%) | 1 ( 0.0%) | 15 ( 0.0%) |  |
| **Referral institution** | Medical facility | 764 ( 4.0%) | 4,391 (10.1%) | 5,155 ( 8.2%) | <0.001 |
|  | Non-medical governmental institution | 1,628 ( 8.6%) | 9,366 (21.5%) | 10,994 (17.6%) |  |
|  | Non-governmental organization | 10,906 (57.3%) | 13,412 (30.8%) | 24,318 (38.8%) |  |
|  | Self-referred | 4,759 (25.0%) | 12,220 (28.0%) | 16,979 (27.1%) |  |
|  | Not specified | 975 ( 5.1%) | 4,195 ( 9.6%) | 5,170 ( 8.3%) |  |
| **Type of medical service provided** | External consultation | 1,123 ( 5.9%) | 6,370 (14.6%) | 7,493 (12.0%) | <0.001 |
|  | Hospitalization | 694 ( 3.6%) | 2,211 ( 5.1%) | 2,905 ( 4.6%) |  |
|  | Emergency | 15,988 (84.0%) | 9,392 (21.5%) | 25,380 (40.5%) |  |
|  | Specialized violence care services | 1,159 ( 6.1%) | 24,604 (56.5%) | 25,763 (41.1%) |  |
|  | Other service | 68 ( 0.4%) | 1,007 ( 2.3%) | 1,075 ( 1.7%) |  |
| **Type of care provider** | Physician | 17,304 (90.9%) | 15,372 (35.3%) | 32,676 (52.2%) | <0.001 |
|  | Psychologist | 979 ( 5.1%) | 21,275 (48.8%) | 22,254 (35.5%) |  |
|  | Social worker | 308 ( 1.6%) | 5,280 (12.1%) | 5,588 ( 8.9%) |  |
|  | Not specified | 441 ( 2.3%) | 1,657 ( 3.8%) | 2,098 ( 3.4%) |  |
| **Post-treatment destination** | Home | 13,707 (72.0%) | 27,522 (63.1%) | 41,229 (65.8%) | <0.001 |
|  | Transfer to another medical unit | 825 ( 4.3%) | 500 ( 1.1%) | 1,325 ( 2.1%) |  |
|  | Violence response service | 540 ( 2.8%) | 10,171 (23.3%) | 10,711 (17.1%) |  |
|  | External consultation | 1,002 ( 5.3%) | 1,205 ( 2.8%) | 2,207 ( 3.5%) |  |
|  | Deceased | 45 ( 0.2%) | 22 ( 0.1%) | 67 ( 0.1%) |  |
|  | Subsequent hospitalization | 1,922 (10.1%) | 1,126 ( 2.6%) | 3,048 ( 4.9%) |  |
|  | Other | 991 ( 5.2%) | 3,038 ( 7.0%) | 4,029 ( 6.4%) |  |
| **Year** | 2015 | 2,960 (15.6%) | 3,949 ( 9.1%) | 6,909 (11.0%) | <0.001 |
|  | 2016 | 3,178 (16.7%) | 4,829 (11.1%) | 8,007 (12.8%) |  |
|  | 2017 | 2,671 (14.0%) | 4,690 (10.8%) | 7,361 (11.8%) |  |
|  | 2018 | 2,390 (12.6%) | 5,635 (12.9%) | 8,025 (12.8%) |  |
|  | 2019 | 1,767 ( 9.3%) | 5,584 (12.8%) | 7,351 (11.7%) |  |
|  | 2020 | 1,468 ( 7.7%) | 4,711 (10.8%) | 6,179 ( 9.9%) |  |
|  | 2021 | 2,144 (11.3%) | 6,514 (14.9%) | 8,658 (13.8%) |  |
|  | 2022 | 2,454 (12.9%) | 7,672 (17.6%) | 10,126 (16.2%) |  |

n: number of patients; %: percentage

**Supplementary Table 6: IPV cases (per 100,000 children and adolescents)**

| **State** | **2015** | **2016** | **2017** | **2018** | **2019** | **2020** | **2021** | **2022** | **Average** |
| --- | --- | --- | --- | --- | --- | --- | --- | --- | --- |
| **Aguascalientes** | 55.2 | 61.7 | 54.7 | 57.3 | 57.5 | 43.7 | 57.5 | 79.3 | 58.4 |
| **Baja California** | 11.3 | 15.2 | 11.8 | 10.6 | 9.6 | 6.6 | 9.7 | 10.7 | 10.7 |
| **Baja California Sur** | 40.9 | 50.9 | 31.3 | 24.6 | 27.1 | 20.5 | 24.6 | 21.7 | 30.2 |
| **Campeche** | 29.8 | 29.1 | 29.4 | 29.8 | 11.8 | 14.4 | 34.8 | 39.9 | 27.4 |
| **Coahuila de Zaragoza** | 29.1 | 31.6 | 31.9 | 26.0 | 15.4 | 37.8 | 56.9 | 69.1 | 37.2 |
| **Colima** | 40.6 | 55.1 | 43.3 | 42.4 | 27.3 | 15.5 | 41.9 | 58.8 | 40.6 |
| **Chiapas** | 7.5 | 9.3 | 8.2 | 9.9 | 13.2 | 10.2 | 20.3 | 20.9 | 12.4 |
| **Chihuahua** | 54.7 | 73.8 | 73.8 | 62.1 | 67.1 | 76.0 | 113.8 | 118.7 | 80.0 |
| **Ciudad de México** | 27.1 | 31.0 | 33.4 | 31.0 | 32.6 | 17.9 | 30.8 | 42.1 | 30.7 |
| **Durango** | 16.4 | 26.6 | 15.5 | 13.5 | 10.2 | 14.7 | 17.1 | 14.7 | 16.1 |
| **Guanajuato** | 71.6 | 88.8 | 88.1 | 92.4 | 79.8 | 75.5 | 79.2 | 95.3 | 83.8 |
| **Guerrero** | 40.0 | 47.2 | 49.4 | 42.5 | 23.6 | 33.3 | 43.4 | 46.1 | 40.7 |
| **Hidalgo** | 69.4 | 84.7 | 73.4 | 87.6 | 73.6 | 53.0 | 105.1 | 115.9 | 82.8 |
| **Jalisco** | 40.4 | 41.0 | 4.8 | 33.4 | 28.4 | 27.2 | 32.2 | 44.0 | 31.4 |
| **México** | 44.1 | 44.9 | 47.4 | 52.0 | 55.4 | 37.6 | 58.3 | 72.2 | 51.5 |
| **Michoacán** | 43.8 | 39.6 | 27.0 | 32.7 | 12.2 | 10.7 | 15.1 | 17.7 | 24.8 |
| **Morelos** | 27.6 | 30.1 | 32.5 | 39.1 | 74.2 | 76.8 | 104.3 | 85.9 | 58.8 |
| **Nayarit** | 11.9 | 21.3 | 27.5 | 29.4 | 11.3 | 4.9 | 13.8 | 18.1 | 17.3 |
| **Nuevo León** | 13.1 | 12.1 | 14.0 | 41.5 | 15.6 | 20.6 | 30.1 | 40.6 | 23.4 |
| **Oaxaca** | 16.6 | 15.1 | 4.4 | 8.0 | 5.8 | 5.2 | 6.9 | 12.0 | 9.2 |
| **Puebla** | 11.5 | 17.9 | 18.2 | 25.2 | 39.1 | 21.2 | 35.2 | 28.7 | 24.6 |
| **Querétaro de Arteaga** | 38.0 | 64.6 | 33.8 | 31.8 | 52.9 | 33.8 | 35.6 | 29.3 | 40.0 |
| **Quintana Roo** | 28.5 | 38.0 | 52.0 | 74.6 | 91.5 | 71.9 | 101.9 | 88.4 | 68.4 |
| **San Luis Potosí** | 65.4 | 80.4 | 77.7 | 86.3 | 70.6 | 62.1 | 83.5 | 96.6 | 77.8 |
| **Sinaloa** | 6.8 | 6.6 | 5.3 | 10.6 | 10.0 | 6.8 | 12.0 | 15.9 | 9.3 |
| **Sonora** | 46.7 | 55.5 | 65.1 | 47.2 | 41.7 | 38.5 | 48.2 | 45.2 | 48.5 |
| **Tabasco** | 47.7 | 55.1 | 56.6 | 41.1 | 39.0 | 26.8 | 49.0 | 70.9 | 48.3 |
| **Tamaulipas** | 17.4 | 18.2 | 26.2 | 24.9 | 31.2 | 15.1 | 20.2 | 34.7 | 23.5 |
| **Tlaxcala** | 53.1 | 45.2 | 96.1 | 64.8 | 51.6 | 16.1 | 38.5 | 43.9 | 51.2 |
| **Veracruz** | 15.3 | 14.0 | 21.0 | 20.3 | 26.4 | 34.4 | 45.3 | 69.2 | 30.7 |
| **Yucatán** | 5.7 | 4.0 | 3.2 | 8.5 | 6.8 | 14.9 | 36.3 | 53.0 | 16.6 |
| **Zacatecas** | 17.3 | 22.4 | 29.4 | 14.2 | 22.0 | 9.5 | 28.2 | 26.1 | 21.1 |

**Supplementary Table 7:** Characteristics of the aggressors of interpersonal violence (IPV) in relation to their victims (Under 5 years old)(n=7,128)

|  |  | **Male**  **3,017 (41.8%)** | **Female**  **4,201**  **(58.2%)** | **Total**  **7,128 (100.0%)** | **p-value** |
| --- | --- | --- | --- | --- | --- |
|  |  | **n (%)** | **n (%)** | **n (%)** |  |
| **Sex of aggressor** | Male | 1,428 (47.3%) | 2,641 (62.9%) | 4,069 (56.4%) | <0.001 |
|  | Female | 577 (19.1%) | 641 (15.3%) | 1,218 (16.9%) |  |
|  | Missing | 1,012 (33.5%) | 919 (21.9%) | 1,931 (26.8%) |  |
| **Age of Aggressor** | Under 18 years | 362 (12.0%) | 550 (13.1%) | 912 (12.6%) | <0.001 |
|  | 18-30 years | 958 (31.8%) | 1,354 (32.2%) | 2,312 (32.0%) |  |
|  | 31-50 years | 450 (14.9%) | 889 (21.2%) | 1,339 (18.6%) |  |
|  | 51-70 years | 67 ( 2.2%) | 195 ( 4.6%) | 262 ( 3.6%) |  |
|  | More than 70 years | 106 ( 3.5%) | 136 ( 3.2%) | 242 ( 3.4%) |  |
|  | Missing | 1,074 (35.6%) | 1,077 (25.6%) | 2,151 (29.8%) |  |
| **Relationship with the victim** | Biological parent | 475 (15.7%) | 742 (17.7%) | 1,217 (16.9%) | <0.001 |
|  | Spouse / partner / boyfriend | 424 (14.1%) | 443 (10.5%) | 867 (12.0%) |  |
|  | Other relative | 38 ( 1.3%) | 257 ( 6.1%) | 295 ( 4.1%) |  |
|  | Stepfather/mother | 568 (18.8%) | 1,052 (25.0%) | 1,620 (22.4%) |  |
|  | Non-family acquaintance | 112 ( 3.7%) | 194 ( 4.6%) | 306 ( 4.2%) |  |
|  | Stranger | 493 (16.3%) | 535 (12.7%) | 1,028 (14.2%) |  |
|  | Other | 352 (11.7%) | 435 (10.4%) | 787 (10.9%) |  |
|  | Not specified | 555 (18.4%) | 543 (12.9%) | 1,098 (15.2%) |  |

n: number of patients; %: percentage

**Supplementary Table 8:** Characteristics of the aggressors of interpersonal violence (IPV) in relation to their victims (5-9 years old) (n=12,219)

|  |  | **Male**  **5,200 (42.6%)** | **Female**  **7,019 (57.4%)** | **Total**  **12,219 (100.0%)** | **p-value** |
| --- | --- | --- | --- | --- | --- |
|  |  | **n (%)** | **n (%)** | **n (%)** |  |
| **Sex of aggressor** | Male | 3,157 (60.7%) | 5,111 (72.8%) | 8,268 (67.7%) | <0.001 |
|  | Female | 833 (16.0%) | 904 (12.9%) | 1,737 (14.2%) |  |
|  | Missing | 1,210 (23.3%) | 1,004 (14.3%) | 2,214 (18.1%) |  |
| **Age of Aggressor** | Under 18 years | 1,470 (28.3%) | 1,228 (17.5%) | 2,698 (22.1%) | <0.001 |
|  | 18-30 years | 1,063 (20.4%) | 1,697 (24.2%) | 2,760 (22.6%) |  |
|  | 31-50 years | 1,123 (21.6%) | 2,159 (30.8%) | 3,282 (26.9%) |  |
|  | 51-70 years | 150 ( 2.9%) | 587 ( 8.4%) | 737 ( 6.0%) |  |
|  | More than 70 years | 153 ( 2.9%) | 179 ( 2.6%) | 332 ( 2.7%) |  |
|  | Missing | 1,241 (23.9%) | 1,169 (16.7%) | 2,410 (19.7%) |  |
| **Relationship with the victim** | Biological parent | 855 (16.4%) | 1,075 (15.3%) | 1,930 (15.8%) | <0.001 |
|  | Spouse / partner / boyfriend | 622 (12.0%) | 638 ( 9.1%) | 1,260 (10.3%) |  |
|  | Other relative | 35 ( 0.7%) | 102 ( 1.5%) | 137 ( 1.1%) |  |
|  | Stepfather/mother | 1,052 (20.2%) | 2,027 (28.9%) | 3,079 (25.2%) |  |
|  | Non-family acquaintance | 164 ( 3.2%) | 694 ( 9.9%) | 858 ( 7.0%) |  |
|  | Stranger | 828 (15.9%) | 780 (11.1%) | 1,608 (13.2%) |  |
|  | Other | 1,042 (20.0%) | 1,071 (15.3%) | 2,113 (17.3%) |  |
|  | Not specified | 602 (11.6%) | 632 ( 9.0%) | 1,234 (10.1%) |  |

n: number of patients; %: percentage

**Supplementary Table 9:** Characteristics of the aggressors of interpersonal violence (IPV) in relation to their victims (10-14 years old) (n=34,234)

|  |  | **Male**  **9,136 (26.7%)** | **Female**  **25,098 (73.3%)** | **Total**  **34,234 (100.0%)** | **p-value** |
| --- | --- | --- | --- | --- | --- |
|  |  | **n (%)** | **n (%)** | **n (%)** |  |
| **Sex of aggressor** | Male | 5,164 (56.5%) | 18,863 (75.2%) | 24,027 (70.2%) | <0.001 |
|  | Female | 897 ( 9.8%) | 2,295 ( 9.1%) | 3,192 ( 9.3%) |  |
|  | Missing | 3,074 (33.7%) | 3,940 (15.7%) | 7,014 (20.5%) |  |
| **Age of Aggressor** | Under 18 years | 2,290 (25.1%) | 3,746 (14.9%) | 6,036 (17.6%) | <0.001 |
|  | 18-30 years | 1,227 (13.4%) | 7,397 (29.5%) | 8,624 (25.2%) |  |
|  | 31-50 years | 2,001 (21.9%) | 7,639 (30.4%) | 9,640 (28.2%) |  |
|  | 51-70 years | 226 ( 2.5%) | 1,418 ( 5.6%) | 1,644 ( 4.8%) |  |
|  | More than 70 years | 387 ( 4.2%) | 513 ( 2.0%) | 900 ( 2.6%) |  |
|  | Missing | 3,005 (32.9%) | 4,385 (17.5%) | 7,390 (21.6%) |  |
| **Relationship with the victim** | Biological parent | 1,062 (11.6%) | 2,688 (10.7%) | 3,750 (11.0%) | <0.001 |
|  | Spouse / partner / boyfriend | 599 ( 6.6%) | 1,224 ( 4.9%) | 1,823 ( 5.3%) |  |
|  | Other relative | 57 ( 0.6%) | 3,519 (14.0%) | 3,576 (10.4%) |  |
|  | Stepfather/mother | 1,071 (11.7%) | 4,703 (18.7%) | 5,774 (16.9%) |  |
|  | Non-family acquaintance | 184 ( 2.0%) | 2,076 ( 8.3%) | 2,260 ( 6.6%) |  |
|  | Stranger | 2,659 (29.1%) | 4,064 (16.2%) | 6,723 (19.6%) |  |
|  | Other | 2,031 (22.2%) | 4,579 (18.2%) | 6,610 (19.3%) |  |
|  | Not specified | 1,473 (16.1%) | 2,245 ( 8.9%) | 3,718 (10.9%) |  |

n: number of patients; %: percentage

**Supplementary Table 10:** Characteristics of the aggressors of interpersonal violence (IPV) in relation to their victims (15-17 years old)(n=62,616)

|  |  | **Male**  **19,032 (30.4%)** | **Female**  **43,584**  **(69.6%)** | **Total**  **62,616**  **(100.0%)** | **p-value** |
| --- | --- | --- | --- | --- | --- |
|  |  | **n (%)** | **n (%)** | **n (%)** |  |
| **Sex of aggressor** | Male | 7,198 (37.8%) | 33,347 (76.5%) | 40,545 (64.8%) | <0.001 |
|  | Female | 496 ( 2.6%) | 2,858 ( 6.6%) | 3,354 ( 5.4%) |  |
|  | Missing | 11,338 (59.6%) | 7,379 (16.9%) | 18,717 (29.9%) |  |
| **Age of Aggressor** | Under 18 years | 1,576 ( 8.3%) | 5,424 (12.4%) | 7,000 (11.2%) | <0.001 |
|  | 18-30 years | 3,560 (18.7%) | 19,732 (45.3%) | 23,292 (37.2%) |  |
|  | 31-50 years | 1,708 ( 9.0%) | 8,428 (19.3%) | 10,136 (16.2%) |  |
|  | 51-70 years | 177 ( 0.9%) | 1,126 ( 2.6%) | 1,303 ( 2.1%) |  |
|  | More than 70 years | 1,253 ( 6.6%) | 861 ( 2.0%) | 2,114 ( 3.4%) |  |
|  | Missing | 10,758 (56.5%) | 8,013 (18.4%) | 18,771 (30.0%) |  |
| **Relationship with the victim** | Biological parent | 591 ( 3.1%) | 2,795 ( 6.4%) | 3,386 ( 5.4%) | <0.001 |
|  | Spouse / partner / boyfriend | 205 ( 1.1%) | 1,277 ( 2.9%) | 1,482 ( 2.4%) |  |
|  | Other relative | 197 ( 1.0%) | 18,157 (41.7%) | 18,354 (29.3%) |  |
|  | Stepfather/mother | 1,197 ( 6.3%) | 4,898 (11.2%) | 6,095 ( 9.7%) |  |
|  | Non-family acquaintance | 119 ( 0.6%) | 1,100 ( 2.5%) | 1,219 ( 1.9%) |  |
|  | Stranger | 9,430 (49.5%) | 6,379 (14.6%) | 15,809 (25.2%) |  |
|  | Other | 2,233 (11.7%) | 5,041 (11.6%) | 7,274 (11.6%) |  |
|  | Not specified | 5,060 (26.6%) | 3,937 ( 9.0%) | 8,997 (14.4%) |  |

n: number of patients; %: percent
